# Lateral Inhibition as the organizer of the bottom-up attentional modulation in the primary visual cortex

**DOI:** 10.1186/1471-2202-16-S1-P243

**Published:** 2015-12-18

**Authors:** Elżbieta Gajewska-Dendek, Andrzej Wróbel, Piotr Suffczynski

**Affiliations:** 1 Department of Biomedical Physics, Institute of Experimental Physics, University of Warsaw, Warsaw, 02-093, Poland; 2 Department of Neurophysiology, Nencki Institute of Experimental Biology, Warsaw, 02-093, Poland

## 

Lateral inhibition is known to occur at all levels of processing of visual information: from retinal ganglion cells, through principal cells in LGN to primary cortical areas [1]. The role of this connectivity module is to contrast lateral variation within the simultaneously processed upstream information.

Our work is based on local field potential (LFP) multichannel signal data [2] recorded from the cats visual cortices activated by attentional tasks: voluntary (top-down) and stimulus driven (bottom-up). These experiments showed that enhanced beta signals (16-24 Hz) serve as a carrier for distributing attentional activation across the visual system. By measuring the correlation strengths between spontaneous LFP signals in different cortical recording sites it was also found that beta activation maps were organized in a mosaic-like pattern within the visual cortex during bottom-up task, whereas top-down task resulted in spatially homogeneous cortical beta excitation.

It was hypothesized that the reason for these different cortical activation patterns was due to lateral inhibition raised by salient visual stimulus during bottom-up task [2]. This work aims to verify a role played by lateral inhibition in producing specific spatial activation patterns found in top-down and bottom-up experimental paradigms by means of computational modelling.

We have constructed a model of neuronal network comprised of single compartment excitatory and inhibitory cells activated according to extended Hodgkin-Huxley dynamics. The network consists of 16 domains representing cortical patches [3], where the neuronal connections keep the anatomical weights, proportions and local structure according to experimental data: neurons are more likely to form synapses with neighboring cells within one domain. Additionally, the domains are more likely to be interconnected with adjacent than with distant ones. LFPs are modelled as a sum of synaptic currents taking into account their distances from a point of measurement.

The modelled neurons receive two kinds of Poisson inputs, which represent the feed-forward input from the thalamus and feed-back activation from higher order cortical regions. The reinforcement of the latter causes widespread increase in beta band power of LFP and represents diffuse, top-down attentional modulating signal. The input from the thalamus arriving to one or more domains represents bottom-up attentional activation of excitatory and inhibitory neurons evoked by salient visual stimulus. The cortical domains activated by this salient input exert additional lateral inhibition on neighboring domains.

Our model shows that the spatial heterogeneity of cortical activation resulting in local decrease of correlation in bottom-up task and homogeneity in top-down task, can be mediated by feed-forward lateral inhibitory influences at the cortical level. The higher cross-correlation variance observed experimentally during bottom-up than in top-down attentional paradigm is also observed in the model.

This modelling study, confirmed that mechanism of lateral inhibition may be sufficient in explaining spatial differences in organization of beta activities in visual cortical areas observed during bottom-up and top-down attentional paradigms.

The network was modelled by means of PyNEST package.
